# Alpha 1 antitrypsin distribution in an allergic asthmatic population sensitized to house dust mites

**DOI:** 10.1186/s13601-018-0231-x

**Published:** 2018-11-02

**Authors:** I. Suárez-Lorenzo, F. Rodríguez de Castro, D. Cruz-Niesvaara, E. Herrera-Ramos, C. Rodríguez-Gallego, T. Carrillo-Diaz

**Affiliations:** 1 Postgraduate and Doctoral School, Universidad de Las Palmas de Gran Canaria, Camino Real de San Roque, 1, 35015 Las Palmas de Gran Canaria, Las Palmas Spain; 2 Pneumology Unit, Hospital Universitario de Gran Canaria Doctor Negrín, Las Palmas de Gran Canaria, Spain; 3 Allergy Unit, Hospital General de Fuerteventura, Puerto del Rosario, Spain; 4 Immunology Unit, Hospital Universitario de Gran Canaria Doctor Negrín, Las Palmas de Gran Canaria, Spain; 5 Allergy Unit, Hospital Universitario de Gran Canaria Doctor Negrín, Las Palmas de Gran Canaria, Spain

**Keywords:** Alpha 1 antitrypsin, Alpha 1 antitrypsin deficiency, House dust mites, Asthma, Allergy

## Abstract

**Background and objective:**

Severe alpha1 antitrypsin deficiency has been clearly associated with pulmonary emphysema, but its relationship with bronchial asthma remains controversial. Some deficient alpha 1 antitrypsin (AAT) genotypes seem to be associated with asthma development. The objective of this study was to analyze the distribution of AAT genotypes in asthmatic patients allergic to house dust mites (HDM), and to asses a possible association between these genotypes and severe asthma.

**Methods:**

A cross-sectional cohort study of 648 patients with HDM allergic asthma was carried out. Demographic, clinical and analytical variables were collected. PI*S and PI*Z AAT deficient alleles of the SERPINA1 gene were assayed by real-time PCR.

**Results:**

Asthma was intermittent in 253 patients and persistent in 395 patients (246 mild, 101 moderate and 48 severe). One hundred and forty-five asthmatic patients (22.4%) with at least one mutated allele (S or Z) were identified. No association between the different genotypes and asthma severity was found. No significant differences in all clinical and functional tests, as well as nasal eosinophils, IgA and IgE serum levels were observed. Peripheral eosinophils were significantly lower in patients with the PI*MS genotype (p = 0.0228). Neither association between deficient AAT genotypes or serum ATT deficiency (AATD) and development of severe asthma, or correlation between ATT levels and FEV1 was observed.

**Conclusion:**

In conclusion, the distribution of AAT genotypes in HDM allergic asthmatic patients did not differ from those found in Spanish population. Neither severe ATTD or deficient AAT genotypes appear to confer different clinical expression of asthma.

## Background

Alpha-1 antitrypsin (AAT) is a serine proteinase inhibitor (PI) that protects alveoli against the destructive effects of neutrophil elastase, proteinase 3 and cathepsin G, which cause destruction of pulmonary parenchyma [1, 2]. Alpha-1 antitrypsin deficiency (AATD) is an autosomal codominant genetic condition first described by Laurell and Erikson in 1963 [3]. Multiple genetic variants in the gene encoding AAT, *SERPINA1*, are associated with low serum AAT levels. The most common deficient alleles are protease inhibitor PI*S and PI*Z, being PI*M the normal variant. Pi*ZZ individuals have severe AAT deficiency, with only 10% of normal serum levels as compared to Pi*MM subjects. Individuals homozygous for the Pi*S (Pi*SS) alleles have approximately 60% of normal serum ATT levels [4, 5]. Although AATD was initially thought of as a rare disease, it has proven to be underdiagnosed in many countries [5, 6]. The distribution of deficient alleles depends on the location; for example, Z variant is more prevalent in North and Western Europe, while S variant has a higher prevalence in the South of Europe, particularly in Spain [7]. Nowadays, different national registries provide the exact prevalence of AATD. However, it is still not considered sufficiently by physicians in the diagnostic phase [8, 9].

Worldwide, AATD has been frequently related to chronic obstructive pulmonary disease (COPD), premature emphysema and liver failure [10–13], but its relationship with asthma remains controversial [14]. AATD is associated with wheezing and dyspnea, which are also characteristic symptoms of asthma. That is why it is sometimes difficult to differentiate between these conditions [14–16] and, according to the American Thoracic Society (ATS)/European Respiratory Society (ERS) and the World Health organization (WHO), diagnosis of asthma is one of the clinical indications for genetic AATD testing [17, 18].

The aim of this study is to analyze the distribution of the most common ATT genotypes in a cohort of asthmatic patients sensitized to house dust mites (HDM). Furthermore, this study attempts to investigate the influence of ATTD and the presence of certain genotypes on the severity of allergic asthma.

## Methods

A cross-sectional cohort study of HDM-sensitive asthmatic patients (skin prick test and specific immunoglobulin E) was carried out. Asthmatic subjects were recruited through the Allergy Clinic of the “Hospital Universitario de Gran Canaria Doctor Negrín” (Canary Islands). All of them were Caucasian, aged over 12. They all had a diagnosis of asthma, with or without rhinitis or other allergic conditions, and complained of typical asthma symptoms such as wheezing, dyspnea and/or other symptoms which they had suffered from over the previous two years. Patients were divided into four groups (intermittent and persistent mild, moderate and severe), according to the severity of their disease and following the Spanish asthma guideline—GEMA4.0 [19]. Written informed consent was obtained from all subjects before participating in the study, which was approved by the Ethics Committee of the hospital.

Asthma was diagnosed on the basis of a history of asthma symptoms and clinical examination (dyspnea, chest tightness, wheezing, cough), and a significant reversibility of their forced expiratory volume in one second (FEV1), as measured with a spirometer (Flowscreen, Viasys, Germany) upon treatment with bronchodilators, at least in one visit during the patient follow-up [19]. Fractional exhaled nitric oxide (FeNO) was also performed (NIOX-MINO^®^ Aerocrine).

Skin-prick tests were performed with several dust mite allergens (*Dermatophagoides pteronyssinus*, *Dermatophagoides farinae, Blomia tropicalis*, *Acarus siro*, *Lepidoglyphus destructor* and *Tyrophagus putrescientae)* from ALK Abelló, Spain. A positive skin-prick test was defined as a mean wheal diameter of at least 3 mm or larger than that of the negative control following current guidelines [20].

In all subjects, blood eosinophils and neutrophils, and total serum immunoglobulin A (IgA), immunoglobulin E (IgE) and specific IgE to *D. pteronyssinus*, *D. farinae, B. tropicalis*, *A. siro*, *L. destructor* and *T. putrescientae* (ImmunoCap, Phadia, Sweden) were determined. ATT serum levels were measured by nephelometry (BNII, Siemens, Erlangen, Germany).

ATT genotypes were determined by using real-time polymerase chain reaction (PCR) and LigthCycler 2.0 for the detection of the mutation according to the technique previously described [21].

Statistical analysis was performed using the nonparametric Kruskal–Wallis test for quantitative variables and Chi square or Fisher tests for qualitative variables. Binary logistic regression was used to determine the association between genotypes and the presence or absence of severe persistent asthma, adjusting for age, smoking habit, significant comorbidity, previous treatment received and some analytical values. The possible correlation between the serum levels of ATT and bronchial functional test parameters was evaluated by Spearman's rank correlation coefficient. A value of p < 0.05 was considered to be statistically significant. All analyses were performed using the R Project (Version 1.0.153) [22].

## Results

During a period of 22 months, 648 asthmatic patients over 12 years (median age 29 years) who were allergic to HDM were recruited into the study. Four hundred and twenty-five (66%) were females and 54 (8%) were smokers. Demographic and clinical characteristics of the patients are listed in Table 1.

**Table 1:** Demographic, clinical and analytical features of the studied population

	Global (n = 648)	Intermittent (n = 253)	Mild (n = 246)	Moderate (n = 101)	Severe (n = 48)	P value*
Sex, n (%)						0.316
Male	233 (34)	90 (36)	84 (34)	38 (38)	11 (23)	
Female	425 (66)	163 (64)	162 (66)	62 (62)	37 (77)	
Age, years						1.72e−12^‡^
Median (IQ_25–75_)	29 (20–38)	27 (20–36)	27 (19–35)	34 (24–44)	41 (34–53)	
Smoker n (%)	54 (8)	24 (9.5)	21 (8.5)	9 (9)	0	0.369
Obesity n (%)	115 (18)	46 (18.2)	36 (14.9)	19 (19.2)	13 (27)	0.221
Conjunctivitis n (%)	107 (16.5)	54 (21.3)	40 (16.3)	11 (11)	2 (4.2)	0.008201^‡^
Rhinitis n (%)	640 (98.8)	250 (98.8)	244 (99.2)	99 (99)	47 (97.9)	0.885
Polyposis n (%)	17 (2.62)	1 (0.8)	2 (0.8)	9 (9)	4 (8.3)	0.00013^‡^
Chronic sinusitis n (%)	32 (4.94)	11 (4.3)	11 (4.5)	8 (8)	2 (4.2)	0.519
Atopic dermatitis n (%)	23 (3.55)	12 (4.7)	5 (2)	5 (5)	1 (2.1)	0.295
Previous pneumonia n (%)	5 (0.77)	1 (0.4)	2 (0.8)	1 (1)	1 (2.1)	0.332
FEV1 (l)						3.98e−22^‡^
Median (IQ_25–75_)	2.9 (2.5–3.4)	3.1 (2.7–3.6)	2.9 (2.6–3.5)	2.6 (2.2–3.1)	2.0 (1.7–2.5)	
FEV1 (%)						1.14e−24^‡^
Median (IQ_25–75_)	90 (81–100)	95 (88–104)	90.5 (84–98)	78 (68–90)	74 (60–81)	
Feno (ppb)						0.873
Median (IQ_25–75_)	40 (23–72)	41 (23–73)	41 (26–72)	35.5 (19–69)	38 (21–60)	
Total IgE (IU/ml)^§^						0.0789
Median (IQ_25–75_)	255 (117–563)	225 (111.5–533)	274 (113–615)	305 (124–711)	213 (93–426)	
IgE Dermatophagoides pteronyssinus						0.0637
Median (IQ_25–75_)	42 (15–100)	34 (13–99)	51 (21–101)	44 (16–100)	42 (5.8–98)	
IgE Dermatophagoides farinae						0.119
Median (IQ_25–75_)	27 (11–79)	21 (10–66)	34 (12–81)	26 (11–85)	32 (5.7–78.8)	
IgE Blomia tropicalis						0.931
Median (IQ_25–75_)	7 (2.1–21)	7 (2–19)	6.8 (3–23)	8.8 (2.1–20)	6.9 (3–19)	
IgE Lepidoglyphus destructor						0.448
Median (IQ_25–75_)	2.4 (1–7)	2.6 (0.9–6)	2.1 (1–6)	2.1 (1–11)	4.1 (2–9)	
IgE Tyrophagus putrescientae						0.743
Median (IQ_25–75_)	2 (1–7.5)	3 (1–7)	2 (1–7)	2 (1–8.5)	3.1 (1–1.9)	
IgE Acarus siro						0.389
Median (IQ_25–75_)	2.6 (1–7)	2.7 (0.9–7.5)	2.4 (1–7)	5 (1–9)	1.3 (0.7–1.8)	
IgA (mg/dL)^§^						0.00024^‡^
Median (IQ_25–75_)	217 (162–279)	220.5 (164–292)	205.5 (157–260)	205.5 (161–271.5)	268 (220–334)	
Eosinophils (10^9^/L)^§^						0.153
Median (IQ_25–75_)	0.3 (0.2–0.5)	0.3 (0.2–0.5)	0.3 (0.2–0.5)	0.3 (0.2–0.5)	0.3 (0.1–0.5)	
Nasal cytology eosinophils (%)						0.265
Median (IQ_25–75_)	37.5 (10–70)	40 (10–70)	40 (15–70)	30 (5–50)	40 (5–60)	
AAT (mg/dL)^§^						0.945
Median(IQ_25–75_)	134 (118–154)	135 (115–159)	134 (118–154)	133 (116–149)	135 (119–144)	

FEV1 (forced expiratory volume in the 1 s), FENO (fractional exhaled nitric oxide), IgE (immunoglobulin E), IgA (immunoglobulin A), AAT (alpha1 antitrypsin)

* Kruskal–Wallis statistic analysis for continuous non normal variables and Chi square or F-test for nominal ones

^‡^Statistical significance, p value < 0.01

^§^Normal value IgE: 10–179 IU/ml; Normal value of IgA: 80–310 mg/dl; Normal value of blood eosinophils: 0–0.54 × 109/L; Normal value ATT: 100–200 mg/dL (1.0–2.0 g/L)

According to the GEMA4.0, 253 asthmatic patients were classified as intermittent and 395 as persistent (246 mild, 101 moderate, and 48 severe). Patients with severe asthma were significantly older (p = 1.72^−12^) as has been previously reported [23]. In addition, a slightly higher percentage of ex-smokers were seen in the group with severe asthma (p = 0.0301), which was considered in the posterior multivariate analysis. Regarding the clinical comorbidity, there was a significantly higher proportion of patients with conjunctivitis in the intermittent asthma group (p = 0.008201) and polyposis in the moderate group (p = 0.00013), while no significance was observed in terms of rhinitis, chronic sinusitis, atopic dermatitis, previous pneumonias or even frequent respiratory infections the year before entering the study. Respiratory function values and analytical determinations are also represented in Table 1.

Median blood eosinophil count was 0.3 × 10^9^/L (0.2–0.5). Median percentage of eosinophils found in the nasal cytology was 37.5% (10–70), with no significant differences among all stages of disease severity. The median serum IgE was high, 255 IU/ml (117–563) as compared to the normal value (< 100 IU/ml), but there was no significant difference among asthma groups. The values of specific IgE were quite similar among all groups. IgA serum levels were significantly higher in patients with severe asthma (268 mg/dl- normal value 80–310 mg/dl) as compared to the other groups (moderate [205.5 mg/dl], mild [205.5 mg/dl] and intermittent [220.5 mg/dl]) (p = 0.00024).

Median AAT value was 134 mg/dl (118-154), within normal range (100–200 mg/dL), and no significant difference was observed according to the severity of the disease. Sixty-six (10.2%) asthmatics had serum AAT levels below the lower limit of normal (< 100 mg/dl) and only three (0.5%) had severe deficiency (< 57 mg/dl) [24].

One hundred and forty-five patients (22.4%) had a deficient AAT genotype. No individual with severe AAT deficiency genotype (PI*ZZ) was identified in our series.

Demographic and clinical characteristics according to PI genotype are shown in Table 2. The distribution of all different severity asthma stages among all AAT genotypes was similar, and no significant difference was observed. However, peripheral eosinophils were significantly lower in the PI*MS group (p = 0.0228). As was expected, the serum levels of ATT were lower in PI*MZ and PI*SZ groups (p = 1.18^−25^). Analytical and functional respiratory test variables are illustrated in Table 3. Finally, we did not find any significant association between AATD and AAT genotypes and the risk of having severe persistent asthma (Table 4).

**Table 2:** Demographic and clinical characteristics according to alpha 1 antitrypsin genotypes

	PI*MM (n = 503)	PI*MS (n = 110)	PI*MZ (n = 15)	PI*SS (n = 14)	PI*SZ (n = 6)	P value
Sex n (%)						0.525
Male	178 (35)	35 (32)	3 (20)	6 (43)	1 (17)	
Female	325 (65)	75 (68)	12 (80)	8 (57)	5 (83)	
Age, years						0.818
Median (IQ_25–75_)	28 (20–38)	31 (23–38)	30 (24–38)	32 (20–36)	20.5 (19–36)	
Smokers n (%)	37 (7.4)	11 (10)	4 (26.7)	2 (14.3)	0	0.0882
Intermittent asthma n (%)	191 (38)	45 (41)	4 (26.7)	9 (64.3)	4 (66.7)	0.134
Mild persistent asthma (%)	194 (38.6)	39(35.5)	6(40)	5(35.7)	2(33.3)	0.981
Moderate persistent asthma n (%)	78 (15.5)	18 (16.4)	4 (26.7)	0	0	0.303
Severe persistent asthma n (%)	39 (7.8)	8 (7.3)	1 (6.7)	0	0	0.958
Polyposis n (%)	9 (1.8)	6 (5.5)	2 (13.3)	0	0	0.104
Chronic sinusitis n (%)	24 (4.8)	3 (2.7)	2 (13.3)	2 (14.3)	1 (16.7)	0.0516
Atopic dermatitis n (%)	17 (3.4)	4 (3.6)	0	1 (7.1)	1 (16.7)	0.271
Previous pneumonia n (%)	4 (0.8)	1 (0.9)	0	0	0	1
> 3infections/year n (%)	4 (0.8)	0	0	0	0	1
Previous treatment: oral glucocorticosteroids n (%)	10 (2)	2 (1.8)	0	0	0	1
Previous treatment: immunotherapy n (%)	38 (7.6)	10 (9.1)	0	0	0	0.804
Previous treatment: omalizumab n (%)	1 (0.2)	0	0	0	0	1

* Kruskal–Wallis statistic analysis for continuous non normal variables and Chi square or F-test for nominal ones

**Table 3:** Analytical and functional respiratory tests according to alpha 1 antitrypsin genotype

	PI*MM (n = 503)	PI*MS (n = 110)	PI*MZ (n = 15)	PI*SS (n = 14)	PI*SZ (n = 6)	P value*
FEV1 (l)						0.23
Median (IQ_25–75_)	2.9 (2.5–3.4)	2.9 (2.4–3.4)	2.9 (2.7–3.3)	3.1 (2.8–3.7)	3.3 (3.2–3.3)	
FEV1 (%)						0.0763
Median (IQ_25–75_)	90 (81–100)	91 (76–101)	92 (84–105)	97 (86–99)	101 (99–111)	
Total IgE (IU/dL)^§^						0.194
Median (IQ_25–75_)	272 (124–576)	207 (74–538)	165 (115–399)	151 (127–646)	169 (90–582)	
IgE Dermatophagoides pteronyssinus						0.372
Median (IQ_25–75_)	45 (17–100)	31 (9–101)	70 (11–101)	34 (20–89)	61 (30–101)	
IgE Dermatophagoides farinae						0.273
Median (IQ_25–75_)	29 (12–80)	17 (6.6–54)	41 (14–79)	24 (8.8–74)	31 (21–84)	
IgE Blomia tropicalis						0.89
Median (IQ_25–75_)	7 (2.1–22)	8 (2.5–19)	6 (5.2–9.6)	9.5 (2–18)	4.5 (1–8.5)	
IgE Lepidoglyphus destructor						0.348
Median (IQ_25–75_)	2 (1–6)	3.1 (1.1–8)	4 (3.6–10)	4 (2–8)	2.2 (2–16)	
IgE Tyrophagus putrescientae						0.409
Median (IQ_25–75_)	2.3 (1–8)	2.3 (1.4–5)	1 (1–1.01)	1.6 (0.7–8)	2.1	
IgE Acarus siro						0.129
Median (IQ_25–75_)	2 (0.9–7)	4 (3–5)	10 (4.3–35)	5.5 (1–11)	23.3	
IgA (mg/dL)^§^						0.333
Median (IQ_25–75_)	216.5 (164–285)	204 (150.8–261)	261 (181–331)	224 (193–238)	255 (160–267)	
Eosinophils (10^9^/L)^§^						0.0228^‡^
Median(IQ_25–75_)	0.3 (0.2–0.5)	0.3 (0.2–0.4)	0.4 (0.3–0.6)	0.5 (0.3–0.6)	0.3 (0.2–0.4)	
Nasal cytology eosinophils (%)						0.378
Median (IQ_25–75_)	40 (10–70)	20 (7.5–60)	45 (33–65)	50 (30–65)	50	
AAT (mg/dL)^§^						1.18e−25^‡^
Median (IQ_25–75_)	138 (123–159)	116 (104–137)	80 (78–87)	89 (86–96)	64 (61.3–70.5)	

FEV1 (forced expiratory volume in the 1 s), IgE (immunoglobulin E), IgA (immunoglobulin A), AAT (alpha1 antitrypsin)

* Kruskal–Wallis statistic analysis

^‡^Statistical significance, p value < 0.05

^§^Normal value IgE: 10–179 IU/ml; Normal value of IgA: 80–310 mg/dl; Normal value of blood eosinophils: 0–0.54 × 109/L; Normal value ATT: 100–200 mg/dL (1.0–2.0 g/L)

**Table 4:** Relationship between AATD or AAT genotypes and severe persistent asthma

	Severe persistent asthma OR (95%CI)	P value*	Multivariable correction**
PI*MM	1.2700 (0.6–2.69)	0.532	0.382
PI*MS	0.9760 (0.44–2.15)	0.953	0.665
PI*SS	0.00000078 (0–Inf)	0.983	0.992
PI*MZ	0.8910 (0.12–6.92)	0.912	0.953
PI*SZ	0.000000791 (0–Inf)	0.989	0.995
SAATD	6.3600 (0.57–71.5)	0.134	0.711
AATD	0.5680 (0.17–1.88)	0.355	0.992

PI: protease inhibitor, SAATD (severe alpha 1 antitrypsin deficiency, < 57 mg/dl), AATD (alpha1 antitrypsin deficit, < 100 mg/dl)

* Binary logistic regression

** Adjusted for age, conjunctivitis, polyposis, ex-smokers, passive smokers

## Discussion

Many authors have suggested an increased risk of asthma with some AAT genotypes. Eden et al. [16] showed that 44% of patients with AATD (20–25% of them with an allergy) had asthma, which was three times more prevalent in PI*MZ subjects than in PI*ZZ individuals. Other investigators have found an even higher percentage of asthmatics among PI*SS population when compared to subjects without deficient alleles [25]. We have not found any clinical association between AATD and AAT genotypes and severity of asthma among HDM sensitized patients. Indeed, the distribution of deficient genotypes among all asthma severity categories was very similar to that described previously in Spanish asthmatic population (Table 5).

**Table 5:** Protease inhibitor genotype distribution in different populations

	PI*MM	PI*MS	PI*MZ	PI*SS	PI*SZ	PI*ZZ
Our population (n = 648)	503 (77.62%)	110 (16.98%)	15 (2.31%)	14 (2.16%)	6 (0.93%)	0
Spanish population [7]	–	1/5 (20%)	1/33 (3%)	1/92 (1.1%)	1/278 (0.36%)	1/3344 (0.03%)
Miravitles et al. study [37]	333 (75.7%)	84 (19.1%)	14 (3.2%)	–	0	0

PI (protease inhibitor)

The balance between normal lung inflammation and repair is a complex process that involves pro- and anti-inflammatory cytokines and the accumulation of inflammatory and immune effector cells [26]. In this work, contrary to previous investigations, we studied a specific group of asthmatic patients sensitized to HDM. It has been proved that mites produce a huge inflammatory reaction in the lung, not only through CD4 + Th2 cells that induce an IgE allergic response, but also through the innate immune system [27]. Different researchers have provided ample evidence that some components of *D pteronyssinus*, such as group 1 allergens (Der p 1), can activate different routes that alter the immune system [28]. Other allergens, such as Der p 3 and Der p 6, also seem to contribute to the HDM allergic response [29]. It is even more intriguing how these allergens can also directly damage the respiratory epithelium by activating mast cell independent of IgE [30].

ATT inhibits neutrophil serine proteases and can regulate the chemotaxis of neutrophils in two different ways: inhibition of IL8-CXCR1 interaction and modulation of ADAM17 activity impeding FcγRIIIb release [31]. The inactivation of ATT by some major components of *D pteronyssinus*, such as Der p 1, has already been proven [32]. The majority of our patients (96.5%) were sensitized to this mite and consequently, the anti-inflammatory action of ATT can be missing in this population. Thus, it is conceivable to think that there could be a possible association between AATD and severity of allergic asthma. However, we have not been able to find a significant association between AATD and severe asthma, as it was reported previously by van Veen et al. in asthmatic patients without a known sensitivity [33]. Neither total IgE nor HDM specific IgE were higher in the most severe asthmatic cases [34]. What we observed is higher serum levels of IgA in patients with severe asthma, as has already been reported [35]. That is why we included serum IgA in the multivariate analysis.

We have also assessed the distribution of different AAT genotypes in our series, which does not differ from the general population in our country [7, 36]. Furthermore, asthmatic carriers of deficient genotypes did not have different clinical expression of asthma, as it was reported before in a non-selected population of asthmatic patients [37] and in a population with severe asthma [33]. AAT serum levels were lower in asthmatic carriers of Z allele [38, 39], but there was no correlation of functional respiratory values neither with serum AAT levels nor AAT genotypes. These results are similar to those reported by others [33, 40]. Nevertheless, another study, conducted with children, suggested that, although low levels of AAT do not enhance the risk of developing asthma, an impaired AAT balance may potentially increase the vulnerability for decrease in lung function and bronchial hyperreactivity in asthmatic children [41].

In contrast to previous reports [37], we have found lower levels of blood eosinophils in PI*MS subjects in comparison to other genotypes. Likewise, the percentage of eosinophils in nasal cytology was also lower in the PI*MS group, though not significantly.

We recognize that our study has some limitations. First of all, we did not predict the required sample size by power calculation, but we strongly believe that this sample of patients truly reflects what happens to the asthmatic population. Moreover, our series is the largest sample studied at the moment. Secondly, we could not find any patient heterozygous for Z allele but this genotype is extremely uncommon and less prevalent in Spain. Finally, we only measured serum ATT levels and we do not know if the local production of ATT by pulmonary epithelial cells and macrophages may balance the low serum ATT levels.

To conclude, we could not find any association between AATD and asthma severity among patients sensitized to HDM. Our findings support what has been reported by others in smaller series of asthmatics. The proportion of asthmatics with deficient AAT genotypes in our series is similar to the proportion in the general population. Although the blood count and nasal eosinophils values seem to be different among the different genotypes, more studies are needed to confirm this due to the scarcity of asthmatic allergic populations with alleles PI*Z.

## Abbreviations

ATT: alpha1 antitrypsin
ATTD: alpha 1 antitrypsin deficiency
COPD: chronic obstructive pulmonary disease
FeNO: fractional exhaled nitric oxide
FEV1: forced expiratory volume in 1 s
FVC: forced vital capacity
HDM: house dust mites
ICS: inhalated glucocorticosteroids
IgA: immunoglobulin A
IgE: immunoglobulin E
LABA: long-acting β2-agonists
PI: protease inhibitor
SABA: short-acting β2-agonists
SATTD: severe alpha 1 antitrypsin deficiency
